# The promises of precision medicine – voices from the Nordics

**DOI:** 10.2340/1651-226X.2025.43987

**Published:** 2025-06-11

**Authors:** Mef Nilbert

**Affiliations:** Institute of Clinical Medicine, Division of Oncology, Lund University and Skane University Hospital Comprehensive Cancer Centre, Sweden and Editor-in-chief Acta Oncologica

**Keywords:** oncology, tumor board, personalised, cancer

Precision oncology holds promises for optimized and individualized treatment strategies with positive effects on quality-of-life and improved outcomes. Molecularly matched therapies generally offer a favorable toxicity profile with less fatigue and hematologic toxicities compared to cytotoxic drugs and offer hope for more years with favorable quality-of-life. Next-generation sequencing of tumor tissue, circulating tumor DNA (ctDNA) analyses, gene expression profiling, and whole genome sequencing have to variable degrees been implemented into clinical routine. Stratified precision oncology, based on diagnosis-specific established and validated biomarkers, has evolved into standard of care with challenges largely related to equity of access to molecular diagnostics. Tumor agnostic precision oncology, based on the identification of targetable alterations for off-label use, is being evaluated in a number of national clinical trials that typically include patients with advanced and refractory cancers.

Like other new developments, precision oncology experiences challenges in clinical implementation. New analytical technologies allow for rapid, high-throughput and increasingly comprehensive tumor profiling, though access to diagnostics varies geographically and clinical decision-making is challenged by the plethora of biological variants identified. Clinical decision-making is challenged by the small-size patient cohorts and the limited evidence to guide treatment recommendations, which has spurred recruitment of new competences involved in precision diagnostics and treatment, establishment of molecular tumor boards, structures for data sharing and plans for long-term monitoring [1, 2]. Additionally, guidelines for precision diagnostics and molecular tumor boards and frameworks for clinical decision-making and trial-matching have been developed [3, 4]. The EU has, through the EU4Health and Horizon Europe programs, funded collaborative initiatives aimed to meet upcoming needs through expanded access to precision oncology, sharing of best practices, development of data sharing models, addressing key barriers such as off-label reimbursement and providing data on cost-effectiveness. These initiatives include the Personalized Cancer Medicine for all EU citizens (PCM4EU), Precision Cancer Medicine Repurposing System Using Pragmatic Clinical Trials (PRIME-ROSE) and the upcoming joint action on Personalized medicine [5].

The potential relevance of molecular alterations across cancer types challenges the previous diagnosis-specific pharmacologic approaches to cancer treatment. The possibility to target molecular alteration outside of the current, diagnosis-specific, label has led to the development of a number of pragmatic trials that aim to develop our understanding of treatment effect and seek new effective treatment options. The Targeted Agent and Profiling Utilization Registry study in the US, the CAnadian Profiling and Targeted agent Utilization tRial study in Canada and the Drug Rediscovery Protocol (DRUP)-trial in the Netherlands serve as a precision trial models with a combined basket and umbrella trial design [6]. The DRUP (NCT02925234) trial opened in 2016 and has to date included more than 2,500 patients and 35 affiliated hospitals in the Netherlands. The trial provides 36 available molecularly matched therapies, and an analysis of the first 500 included patients showed a clinical benefit ratio of 33% [6]. The DRUP concept has inspired the opening of more than 10 such trials in various countries, including the Nordic countries, France, the United Kingdom, Portugal, Estonia, Lithuania and Australia.

The Nordic countries have in several aspects been in the forefront of precision oncology with national precision medicine programs and infrastructure investments linked to clinical trials that match patients to new treatment options. In Norway, the national Infrastructure for Precision Diagnostics Norway (InPRED) linked to the national IMPRESS-Norway (NCT04817956) investigator-initiated, prospective, open-label, non-randomized, combined basket and umbrella trial was launched in 2021. The aim is to facilitate patient access to commercially available targeted anti-cancer therapies, and to describe anti-tumor activity and toxicity of targeted therapies. The IMPRESS-Norway trial is based on molecular genetic profiling, which is reimbursed, or ctDNA analysis and is still recruiting patients. A first, preliminary paper in this journal reported on the inclusion of more than 1,100 patients after 2.5 years [7]. At present, with 4 years of experience, more than 2,750 patients have been included and discussed in the national molecular tumor board. Detection of actionable alterations has led to inclusion in one of the IMPRESS-Norway treatment cohorts in 17% and to treatment in other trials and compassionate use programs in 5% (personal communication Å. Helland, H.E.G. Russnes, G.L. Fagereng, K. Taskén). The trial offers 25 different drugs or drug combinations, resulting in more than 200 different treatment cohorts depending on tumor type, genomic alteration, and drug. As of May 2025, 350 patients have started treatment in IMPRESS-Norway with a disease control rate at 16 weeks of 45%. Preliminary results demonstrate that the InPRED-Norway network makes molecular screening widely available to patients with advanced cancer connected to a national molecular tumor board and to the IMPRESS-Norway trial with 20–25% of the patients offered a matched treatment, about half of whom demonstrate clinical benefit.

In Finland, national precision oncology was developed in 2021 with the aim to implement genomic profiling and precision oncology as standard of care, to secure equity of access to molecular diagnostics and clinical trials and to develop precision oncology through a national DRUP-like trial. With these ambitions, Finland launched the national precision oncology trial FINPROVE (NCT05159245) in 2021 [8]. The trial is open at all five university hospitals and is linked to local and a national molecular tumor board (personal communication K. Jalkanen). In May 2025, about 900 patients have been included. The first evaluation, based on 450 patients, showed that 25% of the patients could be offered molecularly matched treatment. The study has access to more than 15 drugs and provides a precision oncology ecosystem for Finland that contributes to harmonized diagnostics, equity of access, development of data storage and sharing capacities and joint decision-making based on a national molecular tumor board.

In Denmark, the Copenhagen Prospective Personalised Oncology program (NCT02290522) was launched already in 2013. More than 2,000 patients with advanced cancer have been subject to genomic profiling in this prospective, single-center, single-arm open-label study. Actionable targets were identified in 57% of the patients and of these 24% initiated targeted therapy with a total of 274 targeted treatment regimens used during the study period [9]. The overall response (OR) rate was 25%, and presence of ESCAT I/II alterations was found to be associated with improved response rates, progression-free survival and overall survival. This was followed by the national ProTarget trial (NCT04341181), which is a national investigator-initiated phase 2, prospective, non-randomized clinical trial aimed to increase access to molecularly targeted anti-cancer drugs, study safety and efficacy of off-label drug use and to provide new insights in resistance pathways. The trial opened in 2020, runs in eight centers, including all university hospitals, contains about 15 different treatment combinations, and, as of May 2025, has included over 300 patients [10] (personal communication U. Lassen).

Acta Oncologica now publishes the results of the first Swedish, investigator-initiated precision oncology initiative [11]. The MolEcularly Guided Anti-cancer drug off-Label Trial (MEGALiT, NCT04185831) is an exploratory study with the aim to demonstrate feasibility, safety, and clinical benefit of precision oncology in patients with advanced-stage cancer. MEGALIT is a prospective open-label, non-randomized, combined basket and umbrella phase 2 trial performed in the two Comprehensive Cancer Centres in Uppsala and Gothenburg. The trial had four parallel drug baskets: the Mitogen-activated protein kinase kinase (MEK) inhibitor cobimetinib, the poly ADP-ribose (PARP) inhibitor niraparib, the mammalian Target Of Rapamycin (mTOR) inhibitor everolimus, and the PD-L1 blocking antibody atezolizumab. Of the 153 patients in the study, 29% could be allocated to a study drug and 25% started treatment. Tumor response evaluations at 16 weeks showed partial remissions in 6% and a disease control rate of 25%. There were 10 grade ≥ 3 adverse events that could possibly be related to trial drug treatment, none of which were considered serious or unexpected. After a median follow-up of 1.9 years, median overall survival for patients starting who were matched to therapy was 7.4 months, compared to 2.7 months for the 61 untreated patients (hazard ratio [HR]: 0.43; log-rank *P* < 0.0001) though survival was 11.8 months for the 50 patients who received treatment according to physician’s choice (HR 0.55; log-rank *P* = 0.012).

The MEGALIT trial brings several important lessons to Swedish cancer care. Indeed, precision oncology with genomics-matched treatment with everolimus, cobimetinib, niraparib or atezolizumab outside of their approved labeling was found feasible, safe and associated with improved overall survival compared with patients not treated. Yet, the study has significant limitations related to equity of access, timeliness and the number of study drugs available. Precision diagnostics based on tumor biopsies initially provided results within 28 days in only 32% of patients, which later improved to 75% when a ctDNA-based approach was used. Further, a substantial fraction of patients found to carry targetable alterations were not able to start treatment, largely due to late-stage disease and poor performance status. This may, however, be counteracted through early access to precision diagnostics, which allows for strategic treatment planning and screening for clinical trial availability while the patient is in standard therapy. Further, Sweden’s decentralized health care system could in this first precision oncology trial, not achieve participation from all university hospitals, which implies inequitable access and negatively influences access to drugs and inclusion rate.

Current data suggest that at least one in three patients who undergo comprehensive genetic diagnostics have an actionable mutation, with the highest rates in rare and poor-prognosis tumors. The frequency of patients who can be matched to treatment varies from 10 to 50% in different studies with tumor response rates of 25–50%. National and international collaborative networks will be a key development to provide equitable access to precision diagnostics, move testing into earlier disease stages and link targetable alterations to new treatment options to profit from the potential of precision oncology. Acta Oncologica has an ambition to contribute to shaping tomorrow’s oncology. It is against this background that we support the biennial Nordic Cancer Precision Medicine symposium in Oslo, September 15–17, 2025. Please see more at NPCM – Nordic Precision Cancer Medicine. Welcome to participate in this upcoming international precision oncology conference with Nordic signatures and global outreach.

## References

[1] Edsjö A, Holmquist L, Geoerger B, Nowak F, Gomon G, Alix-Panabières C et al. Precision cancer medicine: concepts, current practice, and future developments. J Intern Med. 2023;294(4):455–81. 10.1111/joim.13709 37641393

[2] Horgan D, Tanner M, Aggarwal C, Thomas D, Grover S, Basel-Salmon L et al. Precision oncology: a global perspective on implementation and policy development. JCO Glob Oncol. 2025;11:e2400416. 10.1200/GO-24-00416 39847746

[3] van de Haar J, Roepman P, Andre F, Balmaña J, Castro E, Chakravarty D et al. ESMO Recommendations on clinical reporting of genomic test results for solid cancers. Ann Oncol. 2024;35:954–67. 10.1016/j.annonc.2024.06.018 39112111

[4] Westphalen CB, Boscolo Bielo L, Aftimos P, Beltran H, Benary M, Chakravarty D et al. ESMO Precision Oncology Working Group recommendations on the structure and quality indicators for molecular tumour boards in clinical practice. Ann Oncol. 2025;36:614–25. 10.1016/j.annonc.2025.02.009 40194904

[5] Taskén K, Haj Mohammad SF, Fagereng GL, Sørum Falk R, Helland Å, van Waalwijk van Doorn-Khosrovani SB et al. PCM4EU and PRIME-ROSE: collaboration for implementation of precision cancer medicine in Europe. Acta Oncol. 2024;63:34791. 10.2340/1651-226X.2024.34791 38779910 PMC11332530

[6] Haj Mohammad SF, Timmer HJL, Zeverijn LJ, Geurts BS, Spiekman IAC, Verkerk K et al. The evolution of precision oncology: the ongoing impact of the Drug Rediscovery Protocol (DRUP). Acta Oncol. 2024;63:368–72. 10.2340/1651-226X.2024.34885 38779868 PMC11332463

[7] Puco K, Fagereng GL, Brabrand S, Niehusmann P, Støre Blix E, Samdal Steinskog ES et al. IMPRESS-Norway: improving public cancer care by implementing precision medicine in Norway; inclusion rates and preliminary results. Acta Oncol. 2024;63:379–84. 10.2340/1651-226X.2024.28322 38779911 PMC11332498

[8] Jalkanen KJ, Alanne E, Iivanainen S, Kääriäinen O-S, Tanner M, Auranen A et al. A national precision cancer medicine implementation initiative for Finland. Acta Oncol. 2024;63:395–7. 10.2340/1651-226X.2024.32661 38779937 PMC11332475

[9] Belcaid L, Højgaard M, Tuxen I, Spanggaard I, Lassen U, Robinson L et al. Copenhagen Prospective Personalized Oncology (CoPPO) – impact of comprehensive genomic profiling in more than 2000 patients in a Phase I setting. Ann Oncol. 2025:S0923-7534(25)00158-9. 10.1016/j.annonc.2025.04.004 40246201

[10] Kringelbach T, Højgaard M, Rohrberg K, Spanggaard I, Laursen BE, Ladekarl M, Haslund CA. ProTarget: a Danish Nationwide Clinical Trial on Targeted Cancer Treatment based on genomic profiling – a national, phase 2, prospective, multi-drug, non-randomized, open-label basket trial. Kringelbach et al. BMC Cancer. 2023;23:182. 10.1186/s12885-023-10632-9 36814246 PMC9948467

[11] Ny L, Fagman H, Botling J, Mantovaara L, Asplund P, Karlsson H et al. Feasibility and outcome of genomics-guided treatment selection in advanced cancer – the MEGALiT explorative clinical trial. Acta Oncol 2025;64:742-750. 10.2340/1651-226X.2025.43366 40468525 PMC12160592

